# Pyrethroid Resistance in the Major Malaria Vector *Anopheles funestus* is Exacerbated by Overexpression and Overactivity of the P450 *CYP6AA1* Across Africa

**DOI:** 10.3390/genes9030140

**Published:** 2018-03-02

**Authors:** Sulaiman S. Ibrahim, Nathalie Amvongo-Adjia, Murielle J. Wondji, Helen Irving, Jacob M. Riveron, Charles S. Wondji

**Affiliations:** 1 Vector Biology Department, Liverpool School of Tropical Medicine (LSTM), Liverpool L3 5QA, UK; murielle.wondji@lstmed.ac.uk (M.J.W.); helen.irving@lstmed.ac.uk (H.I.); Jacob.riveron_miranda@syngenta.com (J.M.R.); 2 Department of Biochemistry, Bayero University, PMB 3011, Kano, Nigeria; 3 LSTM Research Unit, Centre for Research in Infectious Diseases (CRID), P.O. Box 13591, Yaoundé, Cameroon; natyadji@yahoo.fr; 4 Centre for Medical Research, Institute of Medical Research and Medicinal Plants Studies (IMPM), P.O. Box 13033, Yaoundé, Cameroon

**Keywords:** *Anopheles funestus*, *CYP6AA1*, overexpression, pyrethroids, bendiocarb, resistance

## Abstract

Resistance to pyrethroids (the ingredients in bed net insecticides) in the major malaria vector *Anopheles funestus* is threatening recent gains in the fight against malaria. Here, we established the role of an over-expressed P450, *A. funestus CYP6AA1* in insecticides resistance. Transcription profiling of *CYP6AA1* across Africa using microarray and quantitative reverse transcription polymerase chain reaction (qRT-PCR) revealed that it is significantly more over-expressed in southern African populations compared to West (Benin) and East African (Uganda). Heterologous expression in *Escherichia coli* coupled with metabolism assays demonstrated that *CYP6AA1* metabolises type I (permethrin) and type II (deltamethrin) pyrethroids, as well as bendiocarb (a carbamate). Transgenic *Drosophila melanogaster* flies over-expressing *CYP6AA1* were significantly more resistant to pyrethroid insecticides, permethrin and deltamethrin compared with control flies not expressing the gene, validating the role of this gene in pyrethroid resistance. In silico modelling and docking simulations predicted the intermolecular receptor-ligand interactions which allow this P450 to metabolise the pyrethroids and bendiocarb. Validation of *CYP6AA1* as a pyrethroid resistance gene makes it possible to monitor the spread of resistance in the field where this P450 is over-expressed. Its potential cross-resistance role makes it necessary to monitor the gene closely to inform control programs on molecular basis of multiple resistance in the field.

## 1. Introduction

The last 15 years have been a turning point in the fight against malaria in sub-Saharan Africa with the malaria intervention tools (vector control and artemisinin-based combination therapies) helping to avert an estimated 663 million cases of malaria [1]. Of these, the scale up in coverage with pyrethroid-impregnated long-lasting insecticidal treated nets (LLINs) [2] and indoor residual spraying (IRS) [3] were the largest contributors (~68% of the cases averted) [1]. Despite this progress, malaria is still endemic across the world with heaviest burden (90%) in WHO African region, where the disease takes the life of a child every two minutes [4]. The reliance on insecticides to control the mosquito vectors has imposed a selection pressure on mosquito vectors, with an escalation in insecticides resistance in the major malaria vectors threatening the success of the current control programs [5]. Widespread resistance and multiple resistance to the major insecticides used in LLINs and IRS by the species from the *Anopheles gambiae* Complex and the *Anopheles funestus* Group are increasingly common [5,6,7]. 

Across Africa, there is a marked heterogeneity in patterns of resistance in different populations of *Anopheles*, even over relatively small distances [8], with available evidences pointing to different molecular mechanisms driving the resistance, even within the same species of *Anopheles* from different localities/regions. For example, in *A. funestus*, the L119F-*GSTe2* mutation is widespread in West Africa where 1,1,1-trichloro-2,2-bis(p-chlorophenyl)ethane DDT resistance is common but absent in southern African populations [9]; and carbamate resistance observed in southern African populations has been attributed to over-expressed P450 *CYP6Z1* and a novel N485I mutation in the *A. funestus acetylcholinesterase-1* gene; a mutation absent in the East and West African populations [10]. This highlights the importance of establishing the spatio-temporal drivers (genes) of resistance in different location/regions to aid in implementing evidence-based control tools and resistance management.

In the absence of *kdr*-type mutations in the voltage-gated sodium channel of *A. funestus,* pyrethroid resistance in this species is mainly metabolic [11]. A handful of P450s, especially from the CYP6 sub-family confers resistance in *A. gambiae s.l.* and *A. funestus* to the four major insecticides used for public health interventions [10,12,13,14]. In the major malaria vector *A. funestus*, the duplicated P450s *CYP6P9a* and *-b* from the major quantitative trait locus (QTL), *rp1* [15] are the principal genes responsible for pyrethroid resistance, across Africa [16,17]. However, the *rp1* QTL which explains 87% of the genetic variance in pyrethroid susceptibility harbours other P450s whose roles have not been validated. These include *CYP6AA1, CYP6P5, CYP6P4a* and *CYP6P2,* consistently shown to be upregulated in multiple resistant populations of *A. funestus* [10,13,18]. These genes need to be functionally characterised before confirming their involvement in pyrethroid resistance. This is notably the case for *CYP6AA1* which exhibited higher over-expression in multiple resistant populations of *A. funestus* from southern Africa compared to *CYP6P4a*, *CYP6P5* and *CYP6P2* [13].

Prior to this study, the actual role of *A. funestus CYP6AA1* in pyrethroid resistance and possible cross-resistance to non-pyrethroid insecticides remain unknown. The ortholog of *A. funestus CYP6AA1* is *A. gambiae CYP6AA1* (which shares 87% identity), and has been shown to be over-transcribed in multiple resistant populations of *A. gambiae,* such as in Burkina Faso [19] and Cameroon [20], but has not been functionally validated as an insecticide metaboliser. However, an ortholog from the Asian malaria vector *A. minimus* (*CYP6AA3*) was shown to be able to metabolise type I and type II pyrethroids [21], and another ortholog *CYP6AA9* was shown to confer resistance to deltamethrin in *Culex pipiens pallens* [22]. 

To fill these important gaps in knowledge, we performed a functional characterisation of *A. funestus CYP6AA1.* Using a combination of heterologous expression and in vitro characterisation, we established that *A. funestus CYP6AA1* metabolises both type I and type II pyrethroids, conferring resistance to the chemicals used for impregnation of bed net insecticides. We validated the ability of *CYP6AA1* to confer pyrethroid resistance in vivo by transgenesis using *Drosophila melanogaster,* with transgenic flies overexpressing the P450 showing significant resistance to pyrethroids compared with control flies. The ability of the *CYP6AA1* to metabolise pyrethroids was further supported by homology modelling and molecular docking simulations which showed that the pyrethroids bind productively in the active site of CYP6AA1 model. In addition, the P450 metabolises the carbamate insecticide bendiocarb, in vitro, though with low activity.

## 2. Materials and Methods

### 2.1. Mosquito Samples

Blood fed, female *A. funestus* resting indoors were collected from three southern African countries: Malawi, Mozambique, and Zambia as described before [23]. Two to five-day old, unfed F_1_ female progenies of the mosquitoes were utilised to test insecticides. This is because age and bloodmeal are known to influence the expression of insecticide resistance genes [24,25]. Multiple resistance profiles of these mosquitoes have been described in a previous publication [13]. The southern African populations were highly resistant to type I and type II pyrethroids, as well as bendiocarb, and moderately resistant to (DDT). In addition, *A. funestus* from Uganda (reported in [26]), from Benin (reported in [9]) and from Ghana (reported in [27]) were also used for comparative expression analysis, and to establish patterns of genetic variability in the DNA sequences. The fully susceptible FANG (Funestus Angola) [28] was used as a reference strain for comparative molecular analyses.

### 2.2. Transcriptional Profiling of CYP6AA1 in Pyrethroid-Resistant Populations

The transcriptional profile of *CYP6AA1* was analysed in a set of microarray data previously published for three southern Africa countries (Malawi, Mozambique and Zambia), and the West African country, Benin using either the 4x44k ((A-MEXP-2245) [16] or the 8x60k (A-MEXP-2374) [13] chips. RNA was extracted separately from three batches, each of 10 female mosquitoes (2- to 5-day-old F_1_ *A. funestus*) from the following groups: (i) resistant (R); (ii) control (C) (mosquitoes not exposed to the insecticide); and (iii) susceptible (S) (mosquitoes from the insecticides susceptible laboratory strain, FANG) and utilised for the microarray analyses. The expression profile of *CYP6AA1* was analysed by comparing field samples that had survived exposure to 0.75% permethrin for 1 h (R) against the fully susceptible laboratory strain, FANG (S). Analysis of data was carried out using GeneSpring GX 12.0 software. To establish differentially expressed genes a cut-off of 2-fold change (FC) and a statistical significance of *p* < 0.01 with Benjamini-Hochberg correction for multiple testing, and q < 0.01 with Storey bootstrapping was applied. To confirm the expression patterns obtained by microarray, quantitative RT-PCR (primers in Table S1) was performed for the *CYP6AA1* gene in Mozambique, Malawi, Zambia, and Benin, as well as Uganda samples, as previously described [13]. The relative expression level and fold change (FC) in permethrin-resistant (R) and control mosquitoes (C) relative to susceptible ones (S) were calculated according to the 2^−ΔΔCT^ method incorporating the PCR efficiency [29] after normalization with the housekeeping genes ribosomal protein S7 (*RSP7*; AGAP010592) and actin5C (AGAP000651).

### 2.3. Amplification and Cloning of Full-Length cDNA of A. funestus CYP6AA1

RNA was extracted using the PicoPure RNA isolation Kit (Arcturus, Applied Biosystems, Foster City, CA, USA) from three batches of 10 multiple resistant mosquitoes from Malawi and Zambia, as well as three batches each of 10 mosquitoes from Uganda and Ghana [13,26,27]. Three batches of the 10 fully susceptible FANG were also extracted. cDNA was synthesised from extracted RNA using SuperScript III (Invitrogen, Waltham, CA, USA) with oligo-dT20 and RNAse H (New England Biolabs, Ipswich, MA, USA). Full-length coding sequences of *CYP6AA1* were amplified separately from each complementary DNA (cDNA) using HotStart II Taq Polymerase (Thermo Fisher Scientific, Waltham, MA, USA) and the primers CYP6AA1_full F and -R, listed in Table S1. In a total, a volume of 14 µL PCR mix made up of 5X Phusion HF Buffer (1.5 mM MgCl_2_ in final reaction), 85.7 µM deoxynucleotide (dNTP) mixes, 0.34 µM each of forward and reverse primers, 0.015 U of Phusion High-Fidelity DNA Polymerase (Fermentas, Waltham, MA, USA) and 10.71 µL of dH_2_0, and 1 µL cDNA was added. Amplification was carried out with the following conditions: 1 cycle at 95 °C for 5 min; 35 cycles of 94 °C for 20 s (denaturation), 57 °C for 30 s (annealing), extension at 72 °C for 90 s; and finally, one cycle at 72 °C for 5 min (final elongation). PCR products were cleaned with a QIAquick^®^ PCR Purification Kit (QIAGEN, Hilden, Germany) and ligated into the pJET1.2/blunt cloning vector using the CloneJET PCR Cloning Kit (Fermentas). These were then cloned into the *E. coli DH5α,* plasmids miniprepped with the QIAprep^®^ Spin Miniprep Kit (QIAGEN), and sequenced on both strands. 

### 2.4. Cloning and Heterologous Expression of Recombinant A. funestus CYP6AA1

*CYP6AA1* cDNA from the most predominant allele from Malawi (with no variation from five different clones sequenced) was prepared for expression following the strategy of Pritchard and colleagues [30], by fusing cDNA fragments from a bacterial *ompA+2* leader sequence with its downstream ala-pro linker to the NH_2_-terminus of *CYP6AA1* coding sequence, in frame with its initiation codon. This was achieved by a PCR reaction using the *ompA* primers given in Table S1. Details of these PCRs have been previously described [13]. The PCR product was cleaned, digested with *Nde*I and *Xba*I restriction enzymes, and ligated into the *Nde*I- and *Xba*I-linearised expression vector pCWori+, to create the expression construct pB13::*ompA+2*-*CYP6AA1.* This construct was co-transformed together with a plasmid bearing *An. gambiae* P450 reductase (pACYC-AgCPR) into *E. coli JM109*. Membrane expression and preparation follows the procedure of Pritchard [31]. Recombinant CYP6AA1 expressed optimally at 21 °C and 150 rpm, 40 h following induction with 0.5 mM δ-aminolevulinic acid (δ-ALA) and 1mM isopropyl β-D-1-thiogalactopyranoside (IPTG) to the final concentrations. Membrane content of the P450 was determined spectrally and cytochrome P450 reductase activity determined using cytochrome *c* reduction assay as established, respectively [32,33].

### 2.5. In vitro Metabolism Assays with Insecticides

Metabolism assays were conducted with permethrin (representative type I) and deltamethrin (type II) pyrethroids, DDT (an organochlorine), the carbamates-bendiocarb and propoxur, as well as malathion (an organophosphate). Protocols for incubations and high-performance liquid chromatography (HPLC) analyses for the above insecticides followed procedures previously published [14,34]. Briefly, using 0.2 M Tris HCL, membrane containing the recombinant CYP6AA1 and AgCPR, reconstituted with cytochrome b_5_ was incubated with 20 µM insecticide for 1 h, at 30 °C and 1200 rpm shaking. After quenching of reaction for 5 min with 0.1 mL ice-cold methanol tubes were centrifuged at 16,000 rpm and 4 °C for 15 min. 100 mL of supernatant was loaded into HPLC vials and injected into isocratic mobile phase (90:10 *v*/*v* methanol:water) with a flow rate of 1 mL/min and wavelength of 226 nm, to quantify pyrethroids by peak separation using a 250 mm C18 column (Agilent, Acclaim^TM^ 120, Dionex, Sunnyvale, CA, USA). Details of the HPLC conditions for the non-pyrethroid insecticides have been given in previous publications [34,35]. All reactions were carried out in triplicate with experimental samples containing the nicotinamide adenine dinucleotide (NADP+) in the NADPH-regenerating buffer and negative control (not containing NADP+). Enzyme activity was calculated as percentage depletion (the difference in the amount of insecticide(s) remaining in the +NADPH tubes [containing the NADP^+^] compared with the negative control, –NADPH) and a t-test used for statistical analysis. 

Steady state kinetic parameters were determined with permethrin and deltamethrin by measuring the rate of reaction for 20 min while varying the substrate concentrations (3–24 µM) in presence of 22.5 pmol recombinant CYP6AA1. Reactions were performed in triplicates both for +NADPH and –NADPH for each concentration. *K_m_* and *V_max_* were established from the plot of substrate concentrations against the initial velocities and fitting of the data to the Michaelis-Menten module using the least squares non-linear regression in the GraphPad Prism 6.03 Software (GraphPad Inc., La Jolla, CA, USA).

### 2.6. Transgenic Expression of A. funestus CYP6AA1 in Drosophila melanogaster Flies and Insecticides Contact Bioassay

To establish whether over-expression of *CYP6AA1* alone can confer resistance to the pyrethroid insecticides, transgenic *D. melanogaster* flies expressing this gene were generated using the GAL4/UAS system. The preparation of the transgenic flies followed the protocols described previously [13,16]. 

Briefly, full-length *CYP6AA1* was amplified from cDNA using the Phusion High-Fidelity DNA Polymerase (Thermo Fisher Scientific, Waltham, MA, USA) and cloned into the pJET1.2/blunt cloning vector (Thermo Fisher Scientific, Waltham, MA, USA). The primers used are listed in Table S1. One predominant clone from Malawi was selected and cloned into the pUASattB vector using primers containing *Bgl*II and *Xba*I restriction sites. Using the PhiC31 system, clones were injected into the germ-line of a *D. melanogaster* strain carrying the attP40 docking site on chromosome 2 [“y^1^w^67c23^; P{CaryP}attP40, “1;2”] by Genetic Services (Sudbury, MA, USA) to generate the transgenic line UAS-CYP6AA1. Ubiquitous expression of the transgene in adult F_1_ progeny (experimental group) was obtained after crossing virgin females from the driver strain Act5C-GAL4 [“y [1] w [*]; P(Act5C-GAL4-w) E1/CyO”,“1;2”] (Bloomington Stock Centre, Bloomington, IN, USA) with UAS-CYP6AA1 males. Similarly, adult F_1_ control progeny (control group) with the same genetic background as the experimental group but without *CYP6AA1* insert were obtained by crossing virgin females from the driver strain Act5C-GAL4 and UAS recipient line males (which do not carry the pUASattB-CYP6AA1 insertion).

For insecticide bioassay, three to five-day old experimental and control female F_1_ flies were used for the contact insecticide assay with 2% permethrin and 0.15% deltamethrin impregnated filter papers prepared in acetone and Dow Corning 556 Silicone Fluid (BHD/Merck, Hesse, Germany). These papers were rolled and introduced into 45 cc plastic vials to cover the entire wall. The vials were plugged with cotton soaked in 5% sucrose. 20–25 flies were placed in each vial, and the mortality plus knockdown was scored after 1 h, 2 h, 3 h, 6 h, 12 h, 24 h and 36 h of exposure to the insecticide. For each insecticide, assays were performed in five replicates and Student’s *t*-test used to compare the mortality plus knockdown between the experimental groups and the control.

### 2.7. Polymorphism Analysis of CYP6AA1 Across Africa

To establish the pattern of genetic variability of *CYP6AA1,* the full-length cDNA from insecticides resistant individuals across three regions of Africa was amplified, as well as from the FANG [28]. Amplification and cloning approach utilised are provided in Section 2.3. Following sequencing of the gene on both strands, polymorphisms were detected through manual examination of sequence traces using BioEdit version 7.2.3.0 [36] and sequence differences in multiple alignments using CLC Sequence Viewer 6.9 (http://www.clcbio.com/). Different haplotypes were compared by constructing a phylogenetic maximum likelihood tree using MEGA 6.06 [37]. Genetic parameters of polymorphism including number of haplotypes (h) and its diversity (H_d_), number of polymorphic sites (S) and nucleotide diversity (π) were computed using DnaSP 5.10.01 [38]. In addition, a haplotype network was built using the TCS program (http://darwin.uvigo.es/software/tcs.html).

### 2.8. Amino Acid Sequence Characterisation of A. funestus CYP6AA1

To identify the features of *CYP6AA1* which could impact its activity, its coding sequence was compared to other closely related P450s. Putative substrate recognition sites 1-6 of *A. funestus CYP6AA1*, *A. gambiae CYP6AA1* (AGAP002862-PA) and *A. minimus CYP6AA3* (GenBank: AAN05727.1) were compared by mapping their amino acid sequences to that of *Pseudomonas putida CYP101A* (P450cam) [39,40]. Structurally conserved regions of the P450s were also predicted using an online tool, CYPED [41].

### 2.9. Homology Modelling and Docking Simulations

To investigate the ability of *CYP6AA1* to interact with the substrate insecticides, a 3D model of this P450 was created using standalone tool EasyModeller [42] and CYP3A4 (PDB: 1TQN) [43] as a template with overall 35% identity. Virtual datasets of ligand insecticides: 1*R*-*cis* permethrin (ZINC01850374), deltamethrin (ZINC01997854), DDT (ZINC01530011) and bendiocarb (ZINC02015426) were retrieved from the library in ZINC^12^ database (https://zinc.docking.org/) [44]. Docking simulations were carried out using Blind Docking Server (http://bio-hpc.ucam.edu/webBD/index.php/entry) with algorithm based on AutoDock Vina. For each ligand, 30 binding poses were generated and sorted according to binding energy and conformation in the model’s active site. Figures were prepared using the PyMOL 1.7 [45]. Non-bonded interactions were predicted using protein-ligand interaction profiler [46].

To compare predicted activities between *A. funestus CYP6AA1* and its ortholog from *A. gambiae*, amino acid sequence of *A. gambiae CYP6AA1* (AGAP002862-PA) was also modelled and molecular docking simulations with the above insecticides carried out as explained above. In addition, intermolecular interactions between the insecticide ligands and *A. gambiae* CYP6AA1 models was also predicted using protein-ligand interaction profiler [46].

### 2.10. Accession Numbers

The DNA sequences of *CYP6AA1* reported in this paper have been deposited in the GenBank database (GenBank KY615238-KY615259). 

## 3. Results

### 3.1. Transcription Profile of CYP6AA1 in Pyrethroid-Resistant A. funestus Across Africa

**Figure 1:**
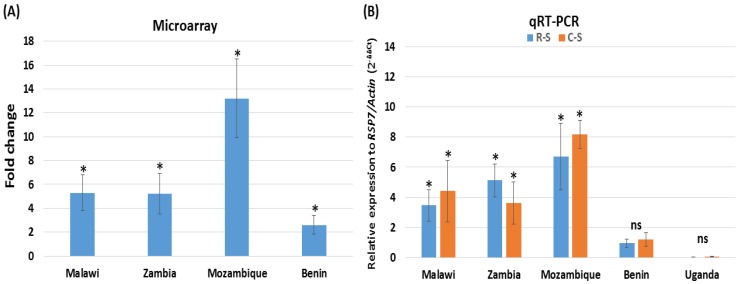
Transcription profile of *CYP6AA1* across Africa. (**A**) Microarray -fold change of *CYP6AA1* in four African countries using the 8x60k chip in comparison to the FANG (Funestus Angola) susceptible strain, from [13]; (**B**) quantitative reverse transcription polymerase chain reaction (qRT-PCR) expression of *CYP6AA1* in five countries comparing the permethrin-resistant mosquitoes to the FANG susceptible (R-S) and the control unexposed to insecticides, to FANG (C-S). * Significantly different at *p* < 0.05. ns = not statistically significant.

Analysis of microarray data revealed that the *A. funestus CYP6AA1* (AFUN015786-RA), was consistently, significantly over-expressed (*p* < 0.05) in the pyrethroid-resistant populations from southern African countries (Malawi, Mozambique and Zambia) compared to the susceptible FANG. The highest fold-change (FC) was observed in Mozambique with FC of 13.2 (Figure 1A) more than twice the level observed in Malawi (FC of 5.3) and Zambia (FC of 5.3), consistent with the higher pyrethroids resistance levels recorded in Mozambique [47]. *CYP6AA1* was also significantly over-expressed in the mosquitoes from Benin (West Africa), compared with FANG, but at a much lower level (FC 2.6). 

qRT-PCR of *CYP6AA1* in these four countries confirmed the microarray expression patterns, with a higher over-expression of *CYP6AA1* in southern Africa (with again the highest level observed in Mozambique) (Figure 1B). Low and non- significant level of expression was observed in Benin for both the permethrin-resistant and the control mosquitoes. The population of Uganda (Tororo) from East Africa was also analysed, but it showed down-expression of *CYP6AA1* compared to the FANG.

### 3.2. Expression Pattern of Recombinant *CYP6AA1*

Optimal expression of recombinant CYP6AA1 was obtained 45 h post-induction, with a P450 content of 0.2 nmol/mg protein ± 0.02 (*n* = 3). This is much lower than the concentrations previously reported for other recombinant P450s, for example *A. funestus* CYP6P9a and CYP6P9b [13]. The recombinant protein produced cytochrome P450 reductase activity of 31.46 nmol cytochrome c reduced/min/mg ± 5.46 (*n* =3), an activity lower than established for CYP6P9a and CYP6P9b [13]. 

### 3.3. Validation of the Role of *A. funestus* CYP6AA1 in Metabolism of Insecticides Using In Vitro Metabolism Assays

**Figure 2:**
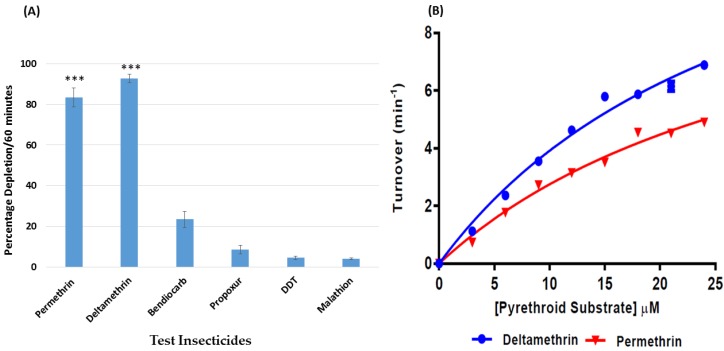
Metabolism of pyrethroids by recombinant *A. funestus* CYP6AA1. (**A**) Percentage depletion of various insecticides (20µM) with recombinant CYP6AA1; results are average of three replicates compared with negative control (-NADPH); *** Significantly different from -NADPH) at *p* < 0.005. (**B**) Michaelis-Menten plot of permethrin and deltamethrin metabolism by recombinant CYP6AA1 protein. Values are mean ± S.E.M. of three experimental replicates compared with negative control, without NADPH (-NADPH).

HPLC analyses established that CYP6AA1 metabolises permethrin and deltamethrin respectively, with high depletion of 83.5% ± 4.6 (*p* < 0.005) and 92.77% ± 2.26 (*p* < 0.001), after an hour of incubation (Figure 2A). No activity was observed towards non-pyrethroid insecticides except for bendiocarb, which exhibited a depletion of 23.42% ± 4.02 (although not statistically significant [*p* = 0.08]), with polar metabolites eluting at the beginning of the chromatogram of incubation with NADPH+. These kind of putative metabolites have been described from in vitro assays of recombinant *A. funestus* CYP6Z1 with bendiocarb [10]. 

In contrast, very low activity was observed with propoxur (8.51% ± 2.11, *p* = 0.2) with no polar metabolites eluting at the beginning of chromatogram. Also, less than 5% of DDT (even following addition of solubilising agent sodium cholate) and malathion were depleted by the recombinant CYP6AA1, indicating lack of enzymatic activity toward the organochlorine and organophosphate insecticides. This is in line with the DDT and malathion susceptibility observed in southern Africa, Mozambique, for example [47,48]. 

Metabolism of pyrethroids follows the canonical Michaelis-Menten pattern with high maximal catalytic rate (*K*_cat_) of 11.99 min^−1^ ± 2.17 and 15.65 min^−1^ ± 2.642, respectively for permethrin and deltamethrin (Figure 2B). The affinity (*K*_m_) for permethrin and deltamethrin were also comparable, 33.62 µM ± 9.180 and 30.01 µM ± 7.915, respectively. Though these *K*_m_ values are within the ranges described for binding and metabolism by insect cDNA-expressed P450s [49], the values are higher than those obtained from *A. funestus* CYP6P6P9a and CYP6P9b [13], and lower than *K*_m_ values obtained from recombinant *A. minimus* CYP6AA3 and CYP6P7 with pyrethroids [21]. Thus, the recombinant CYP6AA1 exhibited catalytic efficiency of 0.36 min^−1^ µM^−1^ ± 0.12 and 0.51 min^−1^ µM^−1^ ± 0.16 respectively, for permethrin and deltamethrin. 

### 3.4. Validation of the Role of CYP6AA1 in Conferring Pyrethroid Resistance in Drosophila Flies Using In Vivo Transgenic Expression

To establish the role of *CYP6AA1* in pyrethroid resistance in *A. funestus* populations, the P450 was expressed in transgenic *Drosophila* flies which were used in bioassays with pyrethroid insecticides permethrin and deltamethrin. Contact bioassays carried out using 2% permethrin and 0.15% deltamethrin established that transgenic flies over-expressing *CYP6AA1* were resistant to pyrethroids with significantly reduced mortalities for both permethrin and deltamethrin compared to control flies. 

**Figure 3:**
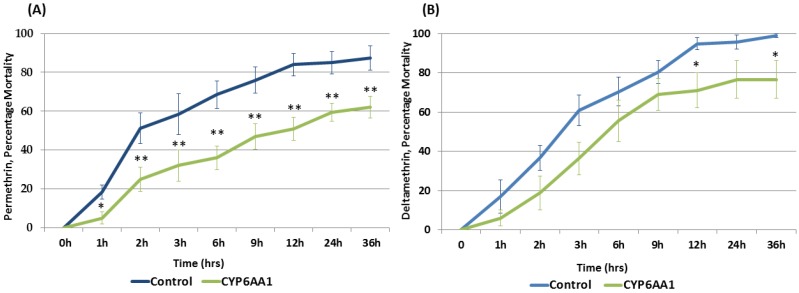
Bioassay results with transgenic flies. (**A**) Progenies of crosses between Actin5C-GAL4 and UAS-CYP6AA1 (transgenic flies over-expressing *An. funestus* CYP6AA1) with permethrin vs. control flies; (**B**) Progenies of crosses between Actin5C-GAL4 and UAS-CYP6AA1 (transgenic flies over-expressing *A. funestus* CYP6AA1) with deltamethrin vs. control flies. Data is shown as mean ± S.E.M. significantly different: * *p* < 0.05, ** *p* < 0.01.

Significantly reduced mortality rates were observed with permethrin for experimental flies (transgenic Act5C-CYP6AA1 females) at all the eight different exposure times compared with the control groups (mean mortality of 38.1% in transgenic Act5C-CYP6AA1 vs. 61.6 in control; *p* < 0.001) (Figure 3A). 

A similar pattern was observed using deltamethrin (Figure 3B) with a significantly reduced mortality in the transgenic Act5C-CYP6AA1 females compared with the control group (mean mortality of 49% in transgenic Act5C-CYP6AA1 vs. 65.3 in control; *p* < 0.05) at 12 h and 36 h of exposure. These results confirmed that over-expression of *CYP6AA1* alone is sufficient to confer resistance to permethrin and deltamethrin. 

### 3.5. Africa-Wide Pattern of Genetic Variability of CYP6AA1

**Figure 4:**
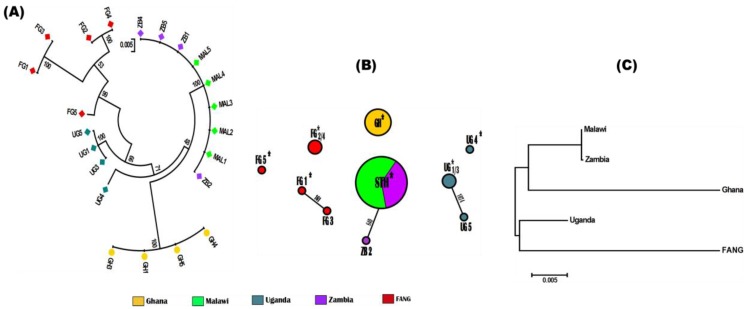
Pattern of genetic variability and polymorphism of *CYP6AA1* DNA sequences across Africa. (**A**) Maximum likelihood phylogenetic tree of *CYP6AA1* DNA sequences; (**B**) Haplotype networks (TCS) for the *CYP6AA1* sequences in *A. funestus*. STH = haplotype shared between Malawi and Zambia; GH = Ghana; UG = Uganda; ZB = Zambia; FG = FANG. Haplotypes are presented in circular shape scaled to reflect their respective frequencies. * = ancestral haplotype. Lines connecting haplotypes represent a single mutation event (respective polymorphic positions are given on each branch). (**C**) Neighbor-joining tree of the genetic distances showing that southern (Malawi and Zambia), West (Ghana) and East (Uganda and FANG) populations are genetically differentiated.

Analysis of the polymorphism patterns of full-length cDNA sequences of *CYP6AA1* (1518 bp) from different regions of Africa revealed relative homogeneity within each geographic region, with haplotypes from each country forming a cluster in the maximum likelihood phylogenetic tree (Figure 4A). However, Malawi and Zambia haplotypes cluster together in the same branch, indicative of their geographic closeness. 

*CYP6AA1* is polymorphic with 10 haplotypes across Africa and 131 polymorphic sites of which 62 were synonymous, and 65 led to amino acids substitutions (Table 1, Supplementary Figure S1). The bulk of the polymorphism were contributed from larger variations in the FANG and Uganda sequences (S = 60 and 24 respectively) compared with the southern African sequences, Malawi with no polymorphism and Zambia (S = 1). The highest homogeneity was observed in southern African countries (especially Malawi with no polymorphism at all and Zambia with only a single polymorphic site) and Ghana (West Africa) with no polymorphism at all, compared with Uganda, with high polymorphism and FANG which exhibited the highest polymorphism. This is also supported by the presence of a predominant haplotype in the southern African populations (Figure 4A and haplotype STH in Figure 4B). Thus, there is reduced variation in the resistant populations from southern Africa and west Africa, and even in Uganda populations compared with the FANG.

Haplotype diversity is high (H_d_ = 0.84), from 10 haplotypes out of 22 sequences from four different countries). The very low H_d_ in the sequences, especially from Malawi and Ghana (H_d_ = 0.00, π = 0.00 for both) suggests a directional selection (selective pressure) acting on *CYP6AA1* in populations from these two regions. A neutrality test of all sequences revealed Li and Fu’s D* as positive but not statistically significant. When all sequences were analysed according to country of origin, statistics was only positive with FANG sequences, suggesting rare polymorphism and lack of background selection. 

Analysis of the genetic structure of the *CYP6AA1* sequences further supported the differences in genetic diversity observed between southern (Malawi and Zambia), West (Ghana) and East Africa (Uganda and FANG) populations. High genetic differentiation estimates were observed between Ghana (0.82 < K_ST_ < 1), East African (0.53 < K_ST_ < 0.68) and the other two populations, whereas the two southern African populations exhibited a null level of genetic differentiation (K_ST_ = 0.0) (Table S2) and cluster together on the Neighbour-joining tree of genetic distances (Figure 4C).

### 3.6. Amino Acid Sequence Characterisation of A. funestus CYP6AA1

**Figure 5:**
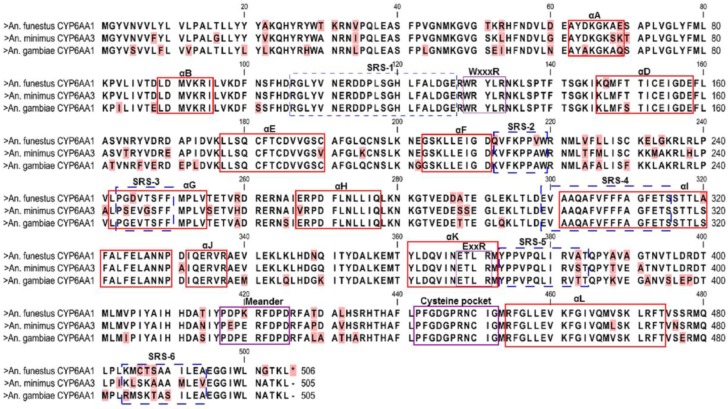
Comparison of *A. funestus* CYP6AA1 amino acid sequences to *A. minimus* CYP6AA3 and *A. gambiae* CYP6AA1. The solid red lines represent helices A-L, while dashed blue lines correspond with the substrate recognition sites 1–6. Solid purple lines identify the structurally conserved motifs of the CYP450s. Variable residues are highlighted in pink. Residues 310–315 corresponds to the oxygen binding pocket.

Comparison of *A. funestus CYP6AA1* to other closely related sequences reveals that it is 89% identical to its ortholog *A. minimus CYP6AA3* (GenBank: AAN05727.1), 87% identical to *A. gambiae CYP6AA1* (AGAP002862) and 57% identical to *Culex pipiens pallens CYP6AA9* (GenBank: AKA45037.1) (Figure 5). Apart from *Culex CYP6AA9* (515 amino acids) all the other three P450s are composed of 505 amino acids.

Sequence-to-sequence mapping reveals that the WxxxR motif, the signatory oxygen-binding pocket (AGFETS)/proton transfer groove, the ExxR motif which stabilises the heme structural core, the cysteine pocket/heme-binding region (PFxxGxxxCxG), which forms the fifth axial ligand to the heme iron [50,51] were all identical and conserved in the three different *Anopheles* sequences (Figure 5). Major sequence variations which could impact the activity of *A. funestus CYP6AA1* compared with *A. minimus CYP6AA3 and An. gambiae CYP6AA1* were observed in the meander, the substrate recognition site 3 (SRS-3), and SRS-6. 

### 3.7. In silico Prediction of Insecticides Binding Parameters and Conformation

**Figure 6:**
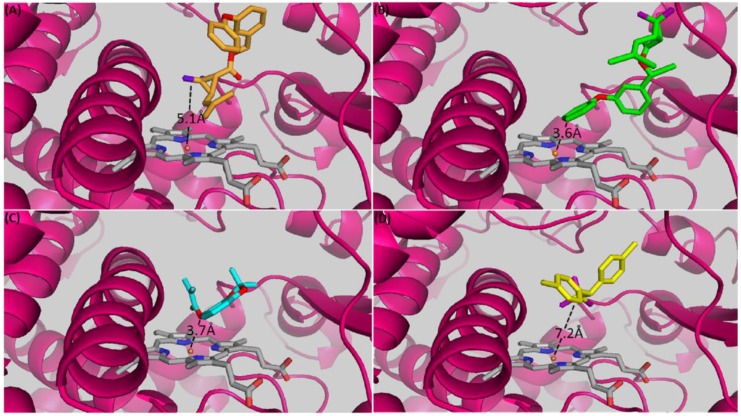
Predicted binding mode of (**A**) permethrin (bright orange stick), (**B**) deltamethrin (green stick), (**C**) bendiocarb (cyan stick), and (**D**) 1,1,1-trichloro-2,2-bis(*p*-chlorophenyl)ethane (DDT) (yellow stick) in *A. funestus* CYP6AA1. CYP6AA1 helices are presented in hot pink; heme atoms are in stick format and grey. Distance between possible sites of metabolism on the insecticides and heme iron are annotated in Angstrom.

To understand the underlying mechanism which makes *A. funestus CYP6AA1* able to metabolise pyrethroid insecticides, a docking simulation was carried out using the homology models of *A. funestus CYP6AA1* and *A. gambiae CYP6AA1* with insecticides from three of the four classes used in public health control of malaria vectors. The binding parameters for each insecticide are provided in Table S3. For *A. funestus* CYP6AA1 model, deltamethrin exhibited the highest affinity (lowest binding energy) with high contribution from hydrophobic interactions and intermolecular hydrogen bonding. The insecticide docked into the active site of CYP6AA1 with the 4′ spot of phenoxy ring oriented above the heme at a distance of 3.6Å from heme iron, suggesting ring hydroxylation (Figure 6B). 4′-hydroxy metabolite has been described as the major route of metabolism of pyrethroids, for example by recombinant CYP6M2 from *A. gambiae* [14] and in other organisms [52]. 

In contrast, lesser but comparable affinity was observed with deltamethrin in the active site of *A. gambiae* CYP6AA1 model consistent with the pose of deltamethrin in the active site of this P450 (*2* position of benzyl ring positioned 6.4Å from heme iron) (Figure S2B). Very high affinity was also observed with permethrin in the two models of CYP6AA1 from both species, but permethrin docked with the gem dimethyl moiety of cyclopropyl group above the heme in both *A. funestus* and *A. gambiae* models, respectively (Figure 6A and Figure S2A). The *trans* methyl group is located at distance of 5.1Å and 5.7Å respectively for the two models, suggesting oxidative attack to produce *trans*-methyl hydroxypermethrin. 

For bendiocarb the second ranked docking solution was the top productive pose in *A. funestus* CYP6AA1. The binding energy of −7.8 kcal/mol indicates moderate affinity to CYP6AA1, compared with *A. gambiae* CYP6AA1 with lower affinity (higher binding energy) of −7.2 kcal/mol. This is consistent with the moderate scores obtained from hydrophobic interactions, which is less than half the values obtained from the pyrethroids. The carbamate insecticide docked productively with the carbamic ester group above the heme catalytic centre, at 3.7Å (Figure 6C), suggestive of ester hydrolysis to generate benzodioxol-4-ol. The same pattern was observed in *A. gambiae* CYP6AA1, but with the carbamate ester located considerably away, at 6.1Å from the heme iron (Figure S2C). 

DDT binds unproductively in the active site of *A. funestus* CYP6AA1 model with the chloride atoms of the trichloromethyl group projecting toward the heme catalytic centre (Figure 6D). The chloride atoms are located 7.2Å from the heme iron, a distance far for meaningful interaction and catalysis to occur. Thus, DDT exhibited the lowest affinity, on average being three times lower than when obtained with the pyrethroids, and half that observed with bendiocarb. Hydrophobic interaction contribution was also the lowest in the case of DDT. However, the trichloromethyl group is pointed away from the heme catalytic centre DDT docked in *A. gambiae* CYP6AA1 far away (9.1Å) from heme catalytic centre (Figure S2D), with no possibility of interactions and catalysis, even though it exhibited strong affinity in this mode (−7.4 kcal/mol). 

Patterns of intermolecular interactions between the ligands and CYP6AA1 models were also established using a protein-ligand interaction profiler [46]. Individual amino acids predicted as responsible for catalysis (involved in hydrophobic interaction, aromatic *π*-stacking and hydrogen bonding) with permethrin, deltamethrin, and bendiocarb were compared from docking simulations of models from *A. funestus* and *A. gambiae,* respectively. For both permethrin and deltamethrin, Phe^309^ was predicted to enhance catalysis through *π*-stacking with the phenoxy ring in the case of permethrin, and with both phenoxy- and benzyl rings in the case of deltamethrin, in *A. funestus* model (Figure S3A,B). This residue belongs to the cluster of three phenylalanine residues (Phe^307^Phe^308^Phe^309^) from the substrate recognition site-4 (SRS-4) within the αI helix (Figure 5). Such an array of aromatic side chains is thought to stabilise the aromatic rings via resonance stabilisation as the alcohol/acid group of the insecticides approach the heme catalytic centre. The same residue along with Tyr^109^ (SRS-1) were predicted to enhance catalysis via *π*-stacking with the aromatic rings of both permethrin and deltamethrin in *A. gambiae* CYP6AA1 active site (Figure S4A,B). For both *A. funestus* CYP6AA1 and *A. gambiae* CYP6AA1 the same residue was also predicted to interact with the aromatic ring of bendiocarb (Figure S3C and S4C, respectively). Another critical residue (Phe^122^ from SRS-1) was predicted to be involved in hydrophobic interactions with aromatic rings of deltamethrin and bendiocarb in the *A. funestus* CYP6AA1 models (Figure S3B,C), and with permethrin, deltamethrin and bendiocarb in *A. gambiae* model (Figure S4A–C). For both models, the side chain of Lys^215^ from SRS-2 was predicted to be involved in salt-bridge to benzyl ring of permethrin and deltamethrin in *A. gambiae* CYP6AA1 and with the benzyl ring of permethrin in *A. funestus* model. 

No hydrogen bonding between permethrin and *A. funestus* CYP6AA1 residues was predicted (Table S3). In contrast, two intermolecular hydrogen bonds were predicted for deltamethrin (Figure S3B), both donated by the guanidinium side chain of Arg^53^ to ester oxygen of the acid moiety. This bond contributed an energy of −1.7 kcal/mol of energy. For bendiocarb, a hydrogen bond was donated by the alcohol group of Thr^314^ in *A. funestus* CYP6AA1 (located within the SRS-4, oxygen binding pocket) to the ester oxygen of the carbamate moiety (Figure S3C). This contributed −1.4 kcal/mol of energy. In contrast, for this insecticide, two hydrogen bonds were predicted in the model of CYP6AA1 from *A. gambiae*: (i) donated by alcohol side chain of Tyr^109^ to benzodioxol moiety; (ii) donated by His^120^ to the amide nitrogen (-NH) of the methylcarbamate moiety. 

Finally, a halogen bond was predicted between Arg^106^ of *A. gambiae* CYP6AA1 with deltamethrin (Figure S4C, in red). This interaction possibly boosts up the affinity towards deltamethrin evident in the lowest energy of binding obtained with this insecticide for *A. gambiae* CYP6AA1. 

## 4. Discussion

The recent disappointment in the efficacy of malaria vaccine RTS_S/AS01 [9] and the fact that scale-up in the distribution of bed nets accounted for 63% of the malaria incidences/cases averted between 2001–2015 [1], suggests that vector control is currently the cornerstone for the control and elimination of malaria. However, timely implementation of the suitable vector control tools relies on the knowledge of insecticide resistance mechanisms and the various genes driving the resistance in the field. 

*A. funestus CYP6AA1* is an important resistance gene, as shown in its consistent upregulation in various populations across Africa, particularly in southern Africa, as it is only moderately over-expressed in West (Benin), and even down-regulated in East Africa (Uganda). Overall, the expression pattern of *CYP6AA1* resembles that of the duplicated P450 genes *CYP6P9a* and *CYP6P9b* which have so far been found to be highly over-expressed in southern Africa and only moderately over-expressed in West (Benin) and East Africa (Uganda and Kenya) [26,53]. *CYP6AA1* is located on the chromosome 2R, together with *CYP6P9a* and *CYP6P9b,* on the same cluster of cytochrome P450s spanning the *rp1* pyrethroid resistance quantitative trait locus (QTL) [15]. 

*CYP6AA1* exhibited pyrethroid activities comparable to values observed from other P450s implicated in pyrethroid resistance in *A. funestus*, notably *CYP6P9a* and *CYP6M7* [13], and *CYP6Z1* [10] or other *A. gambiae* P450s such as *CYP6M2* [14]. The conservation of this gene in *A. funestus*, *A. minimus*, and *A. gambiae* particularly suggests that its detoxification function was retained even after speciation. Indeed, the ortholog from *A. minimus* was shown to metabolise type I and type II pyrethroids in vitro, with higher activity towards deltamethrin than permethrin [21]. The different binding modes of the pyrethroids obtained from molecular docking into active site of *A. funestus* and *A. gambiae* CYP6AA1 suggests possibility of multiple metabolites. Of course, the ortholog *A. minimus* CYP6AA3 is known to possess a very large substrate access channel [54] which accommodates different substrates conformations, resulting in multiple metabolites, e.g., from deltamethrin metabolism [55]. Like *A. funestus CYP6AA1*, the ortholog from *A. minimus* displayed no activity towards malathion and propoxur. The inability of *CYP6AA1* to metabolise malathion further explains the field susceptibility to these insecticides in *A. funestus* [47,56].

It is important to monitor the spread and evolution of this potential cross resistance gene, as it is the second P450 found in *A. funestus* with potential metabolic activity towards bendiocarb, though with very low activity compared with *A. funestus CYP6Z1* [10]. *A. funestus CYP6AA1* is not polymorphic like the *A. funestus CYP6M7* characterised by [13], and is possibly undergoing directional selection in southern and west Africa. This is because it exhibited highest overexpression in the southern Africa, consistent with a very high pyrethroids resistance in the region. It also exhibited the lowest genetic diversity in southern Africa and west Africa, compared to east of Africa and the FANG. It is important to continue monitoring this gene, as it is known that resistance genes could undergo directional selection with beneficial mutations selected [16]. For example, allelic variation of the major resistance genes *CYP6P9a* and *-b* has been shown to be the major driver of resistance to pyrethroid insecticides across Africa in the same *A. funestus* species [17]. 

Though *CYP6AA1* exhibited lower activity in vitro and confers lower resistance to permethrin and deltamethrin in transgenic flies compared with the major pyrethroid resistance gene *CYP6P9b* [17] it is an important enzyme, which possibly works in synergy with the other insecticide resistance genes to orchestrate insecticide detoxification. 

In silico predictions using modelling and docking simulations have become important tools used to study insecticide resistance genes and their heterogeneities in the metabolism of insecticides [14,34,57,58]. The binding and metabolism of permethrin by *CYP6AA1* without hydrogen bonding contributions was as observed with *A. arabiensis* CYP6P4 model [34], and suggests that hydrogen bonding does not contribute to non-bonded interactions to effect catabolism of permethrin in *A. funestus CYP6AA1*. Metabolism of permethrin is possibly driven through hydrophobic interactions, as further supported by the presence of Trp^219^ of SRS-2 within its catalytic hotspot. This is the opposite of the situation with deltamethrin, in which hydrogen bonding plays a significant role in energetic contribution. Indeed, differences in the composition of amino acids lining catalytic hotspot are known to dictate presence or absence of intermolecular hydrogen bonding interactions with resulting differences in choice of substrates even among closely related proteins. For example, polar cages composed of hydrophilic residues in the active site of specialist *CYP6B1* from *Papilio polyxenes* contribute to the P450’s hydrogen bonding capabilities which contribute to its ability to metabolise only two furanocoumarins [59]. In contrast, the generalist *CYP6B8* from *Helicoverpa zea* is devoid of polar cages (hydrogen bonding network) in its active site and was established to metabolise diverse allelochemicals, and even a pyrethroid cypermethrin. 

The inability of *A. funestus* CYP6AA1 to metabolise DDT is consistent with docking predictions, with the organochloride insecticide binding unproductively. Indeed, the productive pose of DDT has been established as C-1 of the trichloromethyl group docking above the heme in *A. gambiae CYP6Z1* [60].

## 5. Conclusions

Knowledge of insecticide resistance in malaria mosquitoes and the mechanisms driving it in the field is essential for design of appropriate malaria control tool and for management of the resistance. However, this requires a thorough understanding of the molecular basis of the resistance to inform control programs and influence policy decisions. Here, we established that *CYP6AA1* contributes to the cocktail of P450s responsible for pyrethroids resistance in southern African populations of *A. funestus*. But, unlike the major pyrethroid resistance genes *CYP6P9a* and *–b*, this P450 exhibited activity toward indoor residual spray insecticide bendiocarb as well, making it a potential cross-resistance gene which should be monitored closely in the field.

## Figures and Tables

**Table 1:** Summary statistics for polymorphism of *CYP6AA1* haplotypes across Africa.

Samples/Country	N	S	h	H_d_	Syn	Nonsyn	π (k)	D (Tajima)	D* (Fu and Li)
Malawi	5	0	1	0.00	0	0	0.00	-	-
Zambia	4	1	2	0.50	0	1	0.0003 (0.500)	−0.6100 ^ns^	−0.4800 ^ns^
Uganda	4	24	3	0.833	18	6	0.0079 (12.00)	−0.8578 ^ns^	−0.8578 ^ns^
Ghana	4	0	1	0.00	0	0	0.00	-	-
FANG	5	60	4	0.900	23	31	0.0215 (32.66)	0.85783 ^ns^	0.9800 ^ns^
All	22	131	10	0.840	62	65	0.026 (40.13)	0.28000 ^ns^	1.0100 ^ns^

N = number of sequences (*n*); S, number of polymorphic sites; h, haplotype; H_d_, haplotype diversity; Syn, Synonymous mutations; Nonsyn, Non-synonymous mutations; π, nucleotide diversity (k = mean number of nucleotide differences); Tajima’s D and Fu and Li’s D statistics; ns, not significant.

## Supplementary Materials

The following are available online at http://www.mdpi.com/2073-4425/9/3/140/s1, Figure S1: Haplotype frequencies and polymorphic sites in *A. funestus CYP6AA1* sequences from across Africa; Figure S2: Predicted binding mode of (A) permethrin (bright orange stick), (B) deltamethrin (green stick), (C) bendiocarb (cyan stick), and (D) DDT (yellow stick) in *A. gambiae CYP6AA1*; Figure S3: Non-bonded interaction between various insecticide structures and *A. funestus* CYP6AA1 active site residues., Figure S4: Non-bonded interaction between various insecticide structures and *A. gambiae* CYP6AA1 active site residues. Table S1: List of primers used in this study; Table S2: Patterns of genetic differentiation between *A. funestus* s.s. populations based on K_ST_ estimates from *CYP6AA1*; Table S3: Affinity and energetic contributions to binding energy of various insecticides docked into the active site of CYP6AA1 models.
