# Supplementary material for: Pyrethroid Resistance in the Major Malaria Vector Anopheles funestus is Exacerbated by Overexpression and Overactivity of the P450 CYP6AA1 Across Africa
Source: Genes (Basel). 2018 Mar 2;9(3):140. doi: 10.3390/genes9030140 (PMC5867861; doi:10.3390/genes9030140)
Supplement: Supplementary file 1 [file genes-09-00140-s001.zip › Suppl Figures Ibrahim et al 2018_genes.pptx]

## Slide 1
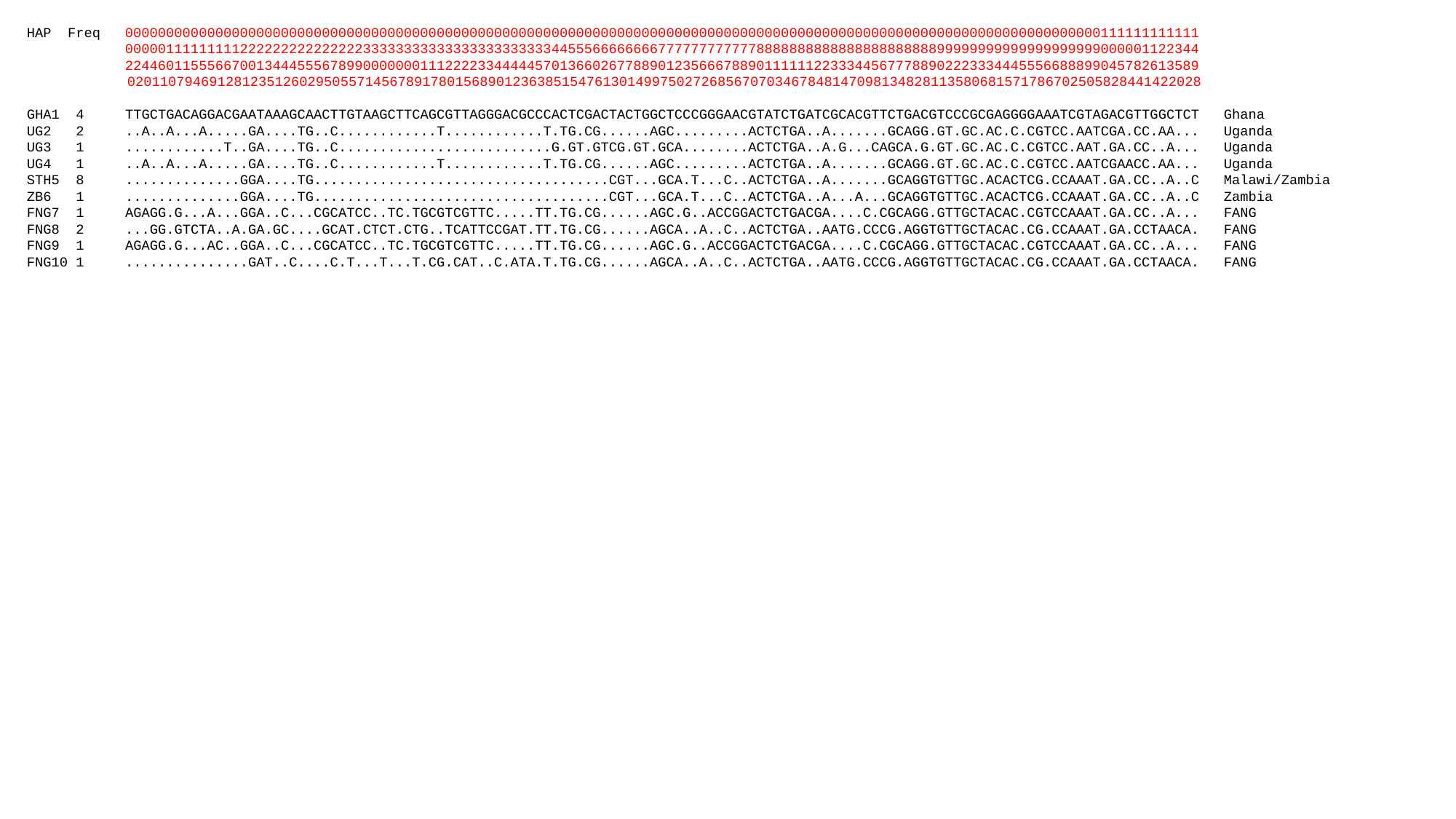

HAP Freq 00000000000000000000000000000000000000000000000000000000000000000000000000000000000000000000000000000000000000000000000111111111111
 00000111111111222222222222222333333333333333333333334455566666666777777777777888888888888888888888899999999999999999999000001122344
 22446011555667001344455567899000000011122223344444570136602677889012356667889011111122333445677788902223334445556688899045782613589
 02011079469128123512602950557145678917801568901236385154761301499750272685670703467848147098134828113580681571786702505828441422028
GHA1 4 TTGCTGACAGGACGAATAAAGCAACTTGTAAGCTTCAGCGTTAGGGACGCCCACTCGACTACTGGCTCCCGGGAACGTATCTGATCGCACGTTCTGACGTCCCGCGAGGGGAAATCGTAGACGTTGGCTCT Ghana
UG2 2 ..A..A...A.....GA....TG..C............T............T.TG.CG......AGC.........ACTCTGA..A.......GCAGG.GT.GC.AC.C.CGTCC.AATCGA.CC.AA... Uganda
UG3 1 ............T..GA....TG..C..........................G.GT.GTCG.GT.GCA........ACTCTGA..A.G...CAGCA.G.GT.GC.AC.C.CGTCC.AAT.GA.CC..A... Uganda
UG4 1 ..A..A...A.....GA....TG..C............T............T.TG.CG......AGC.........ACTCTGA..A.......GCAGG.GT.GC.AC.C.CGTCC.AATCGAACC.AA... Uganda
STH5 8 ..............GGA....TG....................................CGT...GCA.T...C..ACTCTGA..A.......GCAGGTGTTGC.ACACTCG.CCAAAT.GA.CC..A..C Malawi/Zambia
ZB6 1 ..............GGA....TG....................................CGT...GCA.T...C..ACTCTGA..A...A...GCAGGTGTTGC.ACACTCG.CCAAAT.GA.CC..A..C Zambia
FNG7 1 AGAGG.G...A...GGA..C...CGCATCC..TC.TGCGTCGTTC.....TT.TG.CG......AGC.G..ACCGGACTCTGACGA....C.CGCAGG.GTTGCTACAC.CGTCCAAAT.GA.CC..A... FANG
FNG8 2 ...GG.GTCTA..A.GA.GC....GCAT.CTCT.CTG..TCATTCCGAT.TT.TG.CG......AGCA..A..C..ACTCTGA..AATG.CCCG.AGGTGTTGCTACAC.CG.CCAAAT.GA.CCTAACA. FANG
FNG9 1 AGAGG.G...AC..GGA..C...CGCATCC..TC.TGCGTCGTTC.....TT.TG.CG......AGC.G..ACCGGACTCTGACGA....C.CGCAGG.GTTGCTACAC.CGTCCAAAT.GA.CC..A... FANG
FNG10 1 ...............GAT..C....C.T...T...T.CG.CAT..C.ATA.T.TG.CG......AGCA..A..C..ACTCTGA..AATG.CCCG.AGGTGTTGCTACAC.CG.CCAAAT.GA.CCTAACA. FANG

## Slide 2
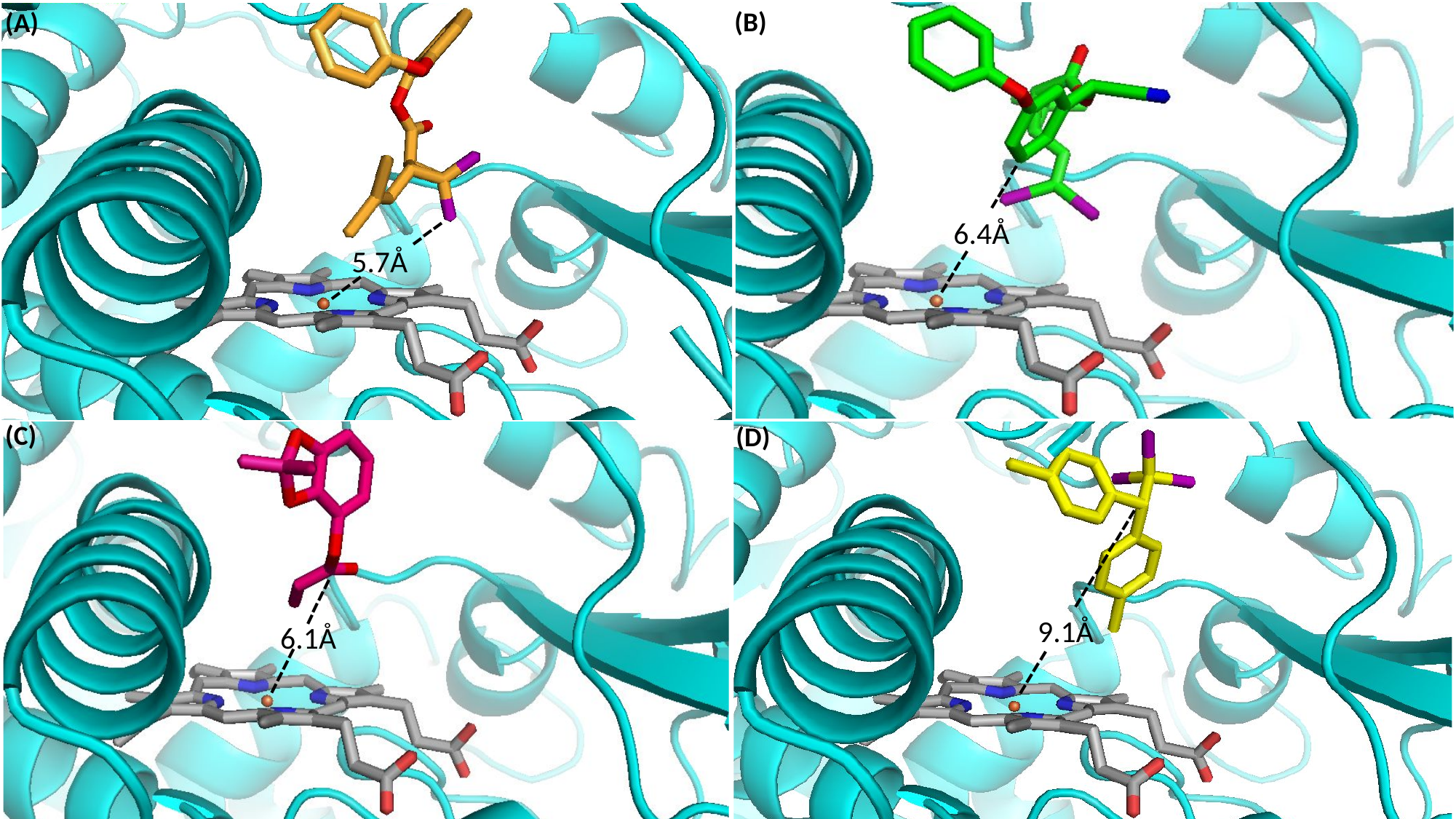

(B)
(A)
----
---
6.4Å
5.7Å
----
---
(C)
(D)
---------
----
9.1Å
6.1Å
----
----

## Slide 3
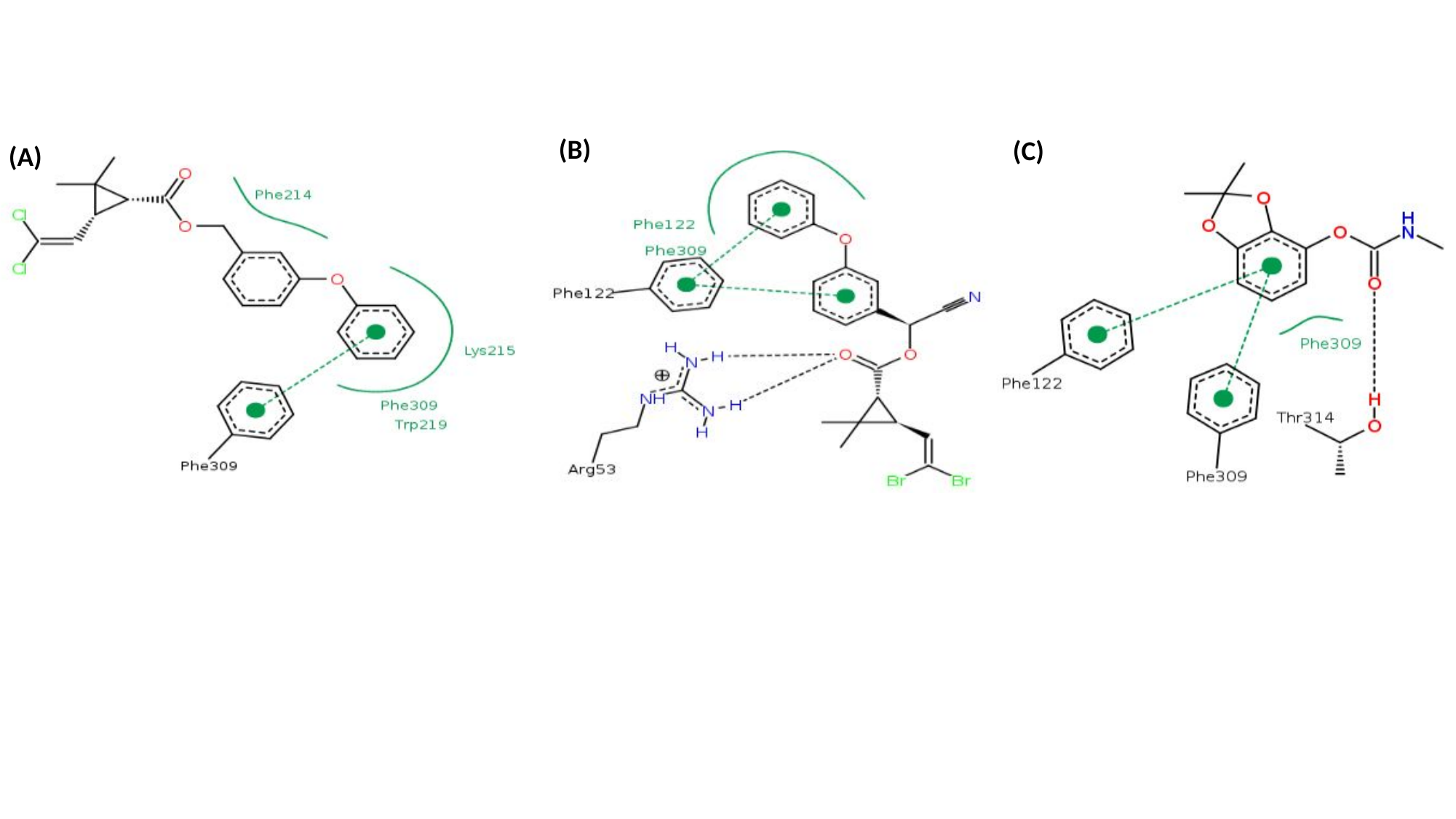

(B)
(C)
(A)

## Slide 4
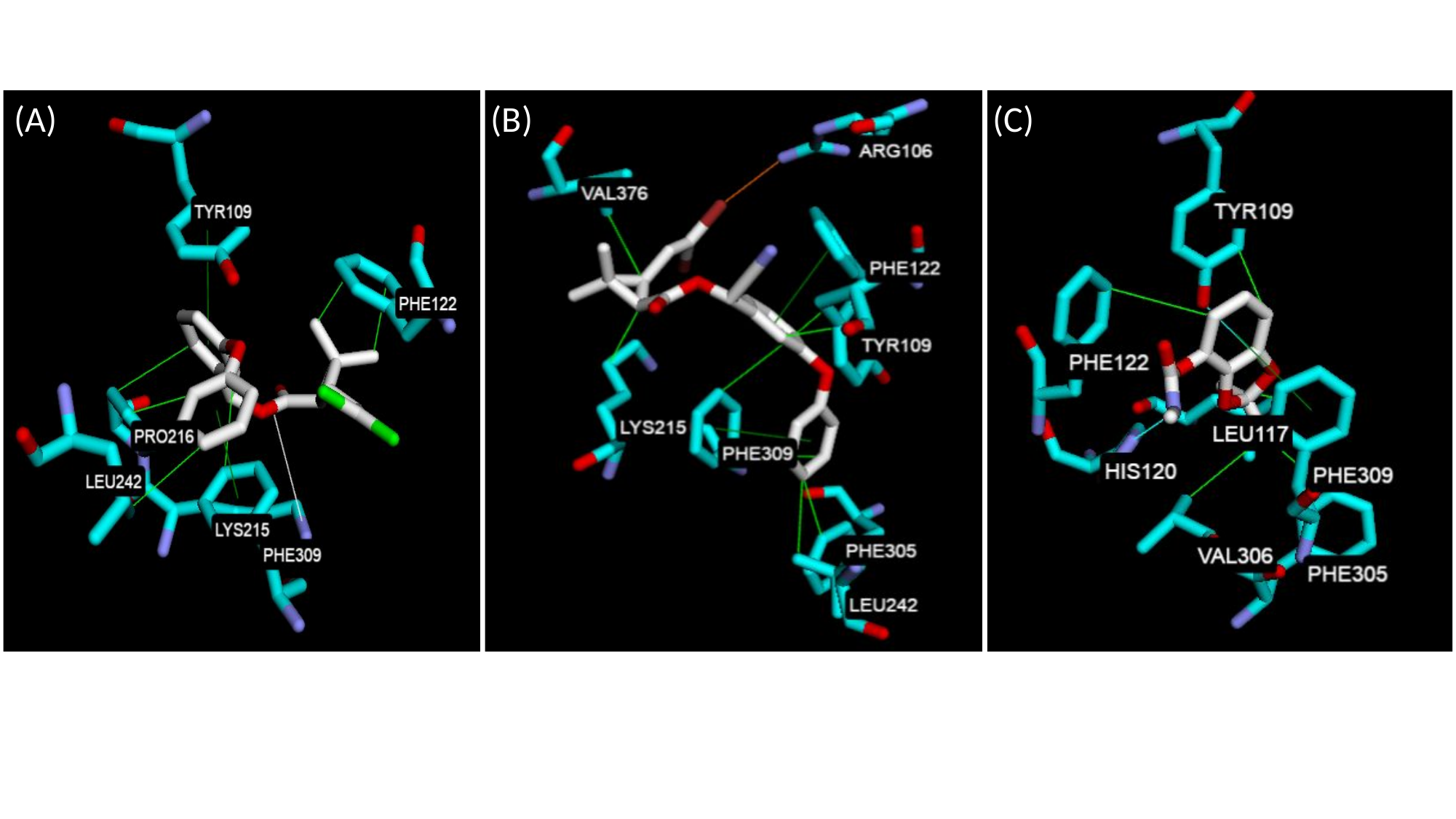

(C)
(B)
(A)
