# Supplementary material for: Pyrethroid Resistance in the Major Malaria Vector Anopheles funestus is Exacerbated by Overexpression and Overactivity of the P450 CYP6AA1 Across Africa
Source: Genes (Basel). 2018 Mar 2;9(3):140. doi: 10.3390/genes9030140 (PMC5867861; doi:10.3390/genes9030140)
Supplement: Supplementary file 1 [file genes-09-00140-s001.zip › SuppI Tables Ibrahim et al 2018_genes.doc]

**Table S1. List of primers used in this study.**

| **Primers used for functional characterisation** | | | |
| --- | --- | --- | --- |
|  | Reverse primer | Forward primer | |
| CYP6AA1full | ATGGAGCTCATTAACGTGGTGTTGGC | TCA CAA TTT TTC CAC CTT CAA GTA ATT ACC CGC | |
| OMPA+2 FORWARD | GGAATTCCATATGAAAAAGACAGCTATCGCG |  | |
| OMPA+2 CYP6AA1F | CAACACCACGTTAATGAGCTCCATCGGAGCGGCCTGCGCTACGGTAGCGAA |  | |
| OMPACYP6AA1R |  | TCTAGAGAATTCTCACACTCTTCTTTCAATCCTC | |
| CYP6AA1_pUASattB *Bgl*II F | TTAGATCTATGGGTTACGTCAACGTGGTGT |  | |
| CYP6AA1_pUASattB *Xba*I R |  | ACTCTAGACTACAGCTTAGTGCCATTCAGC | |
| **Primers used for qRT-PCR validation of transgenes** | | | |
| *CYP6AA1* | ACGACGGTACGCTAACGACT | | TAACGCCAGCTCATACAACG |
| RSP7*(AGAP010592)* | GTGTTCGGTTCCAAGGTGAT | | TCCGAGTTCATTTCCAGCTC |
| Actin *(AGAP000651)* | TTAAACCCAAAAGCCAATCG | | ACCGGATGCATACAGTGACA |

Restriction sites are in different colours and underlined: Green is *EcoR*I, Purple is *Nde*I, Brown is *Bgl*II and Red is *Xba*I site

**Table S2. Patterns of genetic differentiation between *An. funestus* s.s. populations based on K_ST_ estimates from *CYP6AA1*.**

|  | **FANG** | **Ghana** | **Uganda** | **Malawi** |
| --- | --- | --- | --- | --- |
| Ghana | 0.68** (0.06) |  |  |  |
| Uganda | 0.41** (0.19) | 0.82* (0.03) |  |  |
| Malawi | 0.57** (0.10) | 1** (0.00) | 0.68** (0.07) |  |
| Zambia | 0.53** (0.11) | 0.99* (0.00) | 0.65* (0.08) | 0.0^ns^ |

PERMTEST calculates Hudson’s K_ST_ statistic of genetic differentiation. K_ST_ is equal to 12KS/KT, where KS is a weighted mean of K1 and K2 (mean number of differences between sequences in subpopulations 1 and 2, respectively) and KT represents the mean number of differences between two sequences regardless of their subpopulation. *, 0.01 < P < 0.05; **, 0.001 < P < 0.01; ns, not significant.

**Table S3. Affinity and energetic contributions to binding energy of various insecticides docked into the active site of CYP6AA1 models**

| **Insecticide** | **Productive Pose Rank** | **Binding Energy (Kcal/mol)** | **Hydrophobic interaction** | **Hydrogen bond** | **Repulsion (Kcal/mol)** |
| --- | --- | --- | --- | --- | --- |
|  |  | *An. funestus* CYP6AA1 |  |  |  |
| Permethrin | 1^st^ | -10.2 | -3.9 | 0.00 | 1.7 |
| Deltamethrin | 1^st^ | -10.8 | -3.8 | -1.7 | 1.8 |
| Bendiocarb | 2^nd^ | -7.8 | -1.6 | -1.4 | 1.9 |
| DDT | 1^st^ | -4.4 | -1.1 | 0.00 | 1.2 |
|  |  |  |  |  |  |
|  |  | *An. gambiae* CYP6AA1 |  |  |  |
| Permethrin | 1^st^ | -10.4 | -4.3 | 0.00 | 1.0 |
| Deltamethrin | 1^st^ | -10.5 | -4.4 | -0.4 | 1.6 |
| Bendiocarb | 1^st^ | -7.2 | -1.8 | -1.3 | 1.3 |
| DDT | 1^st^ | -7.4 | -2.4 | 0.00 | 0.5 |
